# Case report: Urgent liver pathologies: All in one

**DOI:** 10.3389/fsurg.2022.940856

**Published:** 2022-07-20

**Authors:** Goran Pavlek, Ivan Romic, Kristina Juzbasic, Ana Marija Alduk, Igor Petrovic, Rudolf Radojkovic, Dario Grbavac, Hrvoje Silovski

**Affiliations:** Department of Surgery, University Hospital Centre Zagreb, Zagreb, Croatia

**Keywords:** HCC, rupture, abscess, cyst, hematoma

## Abstract

Ruptured hepatocellular carcinoma (HCC) is a well-known serious complication of this most common primary liver malignancy. However, when HCC rupture is associated with other focal liver lesions, the diagnosis and therapy may be very challenging. Correct differentiation of focal liver lesions is of paramount importance for successful treatment. The aim of this report is to present a unique case of HCC rupture complicated with liver abscess, hematoma and portal vein thrombosis. We discuss possible pathophysiological mechanisms and radiologic findings of such clinical scenarios and review literature related to the management of HCC rupture.

## Introduction

Hepatocellular carcinoma (HCC) is the most common primary liver cancer (90% of cases), representing the sixth leading cause of cancer and the third leading cause of cancer-related mortality. The annual recurrence rate of HCC after surgical resection is 10% and reaches approximately 70%–80% after 5 years (1). Patient prognosis depends on the tumor stage at the time of diagnosis and the possibility of providing radical treatment. Patient stratification and treatment allocation are based on tumor stage, liver function, and performance status. Treatment options include surgical resection, liver transplantation, percutaneous local radiofrequency ablation, transarterial chemoembolization and palliative care. Beside a relatively poor prognosis for large (>5 cm) HCCs, one of the main concerns for clinicians is the risk of HCC rupture which presents a life-threatening condition. Unlike traumatic injuries which may trigger rupture of focal liver lesions such as hemangiomas or HCC, spontaneous rupture occurs with no obvious cause. The reported incidence of spontaneous HCC rupture is increasing and reported to be 3%–26% (2).

Historically, traditional chemotherapy agents have not shown great efficacy in the treatment of HCC, especially when used in an advanced stage of the disease. Therefore, it is still controversial whether adjuvant treatment should be employed after curative hepatectomy for HCC. Current studies are based on novel oncologic therapy which could be beneficial in reducing the recurrence rate of HCC. In the past decade, targeted molecular therapies such as monoclonal antibodies and immune checkpoint inhibitors such as Anti-VEGF antibodies (bevacizumab) have made ground-breaking progress in the treatment of advanced or metastatic HCC (3–5). Deregulation of the PI3K/Akt/mTOR pathway is increasingly implicated in HCC carcinogenesis and it occurs in approximately 45% of HCCs. Inhibitors that target this pathway are a hot topic in HCC management today (6, 7). In addition, Neuropilin 1 (NRP1) is a transmembrane glycoprotein that acts as a multifunctional receptor for various members of the vascular endothelial growth including HCC growth and vascular remodeling. This fact directed recent studies to research the possibility of therapeutically targeting NRP1 for the treatment of HCC (8). Some of these inhibitors have been tested in HCC models, while others are currently undergoing clinical assessment. Several studies using combinations of mTOR inhibitors with conventional chemotherapy, radiotherapy, antimicrotubule agents, as well as other molecular targeting agents, have shown promising antitumor effects in advanced HCC (3–5, 7, 9–12). Most promising therapeutic novelties are related to combination of immunotherapy and antiangiogenic agents. Preclinical studies showed that the immunomodulatory effects of antiangiogenic therapy may be potentiated by concurrent immunotherapy, and this has been well illustrated in several clinical trial data. These results have led to FDA approvement of the combination of atezolizumabd to sorafenib or lenvatinib only. The role of such therapy in the neoadjuvant setting and long-term results after curative resection should yet be investigated. In every case, target and immunotherapy, better patient selection based on molecular tumor characteristics, and the adoption of more precise imaging techniques are almost certain to provide further advances in the treatment of HCC (13, 14).

## Case presentation

**Figure 1:**
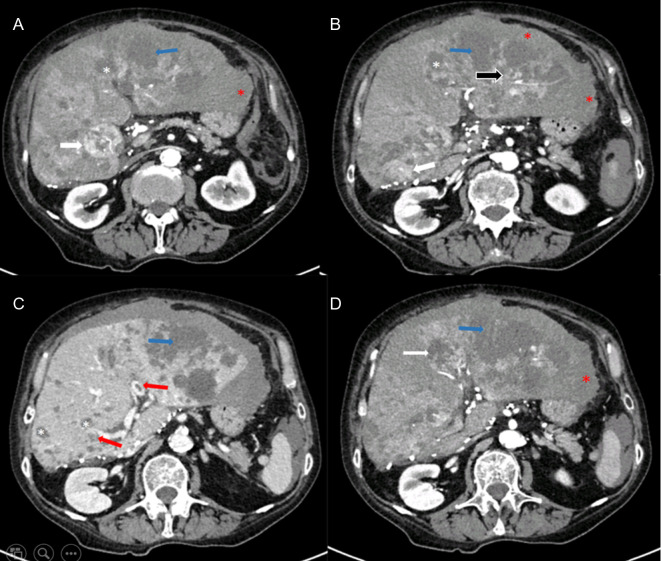
Abdominal axial CT images of the liver in arterial (**A** and **B**) and venous phase (**C** and **D**) showing multiple liver lesions of various characteristics (white arrow-HCC; black arrow- ruptured HCC; white asterisk-simple liver cysts; blue arrow-abscess; red asterisk-hematoma; red arrow-portal vein thrombosis).

**Figure 2:**
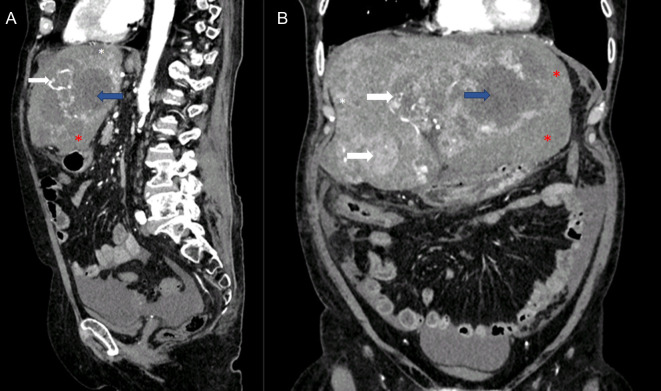
Abdominal frontal (**A**) and sagittal (**B**) CT images showing recurrent HCC (white arrow), liver hematoma (red arrow) and multiple liver abscesses (blue arrow).

A 64-year-old female patient with a medical history of Hepatitis C and radically resected hepatocellular carcinoma (right lateral sectionectomy two years previously) presented to the emergency department with upper abdominal pain, mild fever, and lightheadedness for the last 48 h. The patient had a history of diabetes mellitus for the past 9 years (on insulin therapy for the last two years) and arterial hypertension for the last 12 years. Primary HCC was located in segment VII with the size of 3 cm in diameter. There were no symptoms of increased portal venous pressure or hyperbilirubinemia. Therefore, the patient had initial stage (stage A) disease according to the BCLC staging system. Ultrasound 6 months prior to admission showed no signs of HCC recurrence. Physical examination revealed a moderately tender and distended abdomen, blood pressure of 90/60 mmHg, heart rate of 120/min and fever of 38.8C. Laboratory investigations indicated severe microcytic anemia: hemoglobin of 69 g/L (normal range 121–151 g/L) and hematocrit of 33.2% (normal range 36%–44%) and high transaminase levels, AST 612 U/I (normal range 8–33 U/I) and ALT 432 UI (normal range 7–55 U/I). In addition, elevated inflammatory markers were detected: leukocyte count of 18,500/mm^3^ (normal range 4,500–11,000 mm^3^) and CRP of 211 mg/L (normal range >5 mg/L). An urgent 3-phase abdominal CT scan was performed and it revealed multiple liver lesions of various characteristics, a subcapsular hematoma and a small amount of intraperitoneal ascites (Figures 1, 2).

White arrow is indicating lesions in segments V/VIII and IV with hypervascular pattern and arterial enhancement indicating HCC recurrence. Black arrow shows a similar lesion in segment II/III which was surrounded with hematoma. White asterisk is indicating previously known multiple cystic formations with imperceptible wall suggestive of simple liver cysts. Blue arrow is demonstrating peripherally enhancing and centrally hypoattenuating lesions characteristics of an abscess. Red asterisk is indicating intrahepatic hematoma in the left liver lobe extending subcapsulary and intraperitoneally. In addition, partial thrombosis (70% of vein lumen) with a length of 2 cm of both main portal branches was observed, as showed by red arrow.

**Figure 3:**
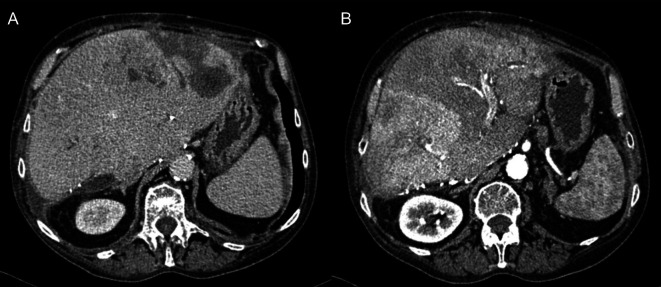
Abdominal CT scan after 12 months in venous (**A**) and arterial phase (**B**) showing slight progression of multicentric HCC nodules with regression of hematomas and abscesses.

The case was discussed at the MDT which concluded that there is no possibility of radical liver resection, but suggested that systemic and locoregional therapy should be initiated after the management of bleeding and portal vein thrombosis since two HCC nodules with diameters greater than 3 cm were present (BCLC stage C-advanced). Conservative treatment was initiated over the next 24 h with blood transfusions (6 doses of red blood cells), antibiotics, and coagulopathy correction, but due to clinical deterioration (hypotension 80/40 and persistently low hemoglobin levels of 65–75 g/L), tratranscatheterterial embolization of branches for segments 2 and 3 was performed. After 48 h the patient became hemodynamically instable again, so surgical exploration was performed which revealed an old intraperitoneal hematoma and moderate active bleeding from the liver edge of segment 3 where the HCC rupture was present. Partial resection of the deteriorated liver parenchyma, hematoma evacuation and drainage of liver abscesses was performed. The patient received 4 pints of fresh blood. In the postoperative period, the red blood count was stabilized (hemoglobin of 105–115 g/L). The postoperative course was uneventful, the patient recovered and was discharged on postoperative day 11 (Table 1). Three months later, transarterial chemoembolization was performed which was followed by chemotherapy (sorafenib) over the next 9 months. At 12 months follow-up, the patient is alive, has no significant disease progression and no signs of liver failure (Figure 3).

**Table 1:** Laboratory results over the treatment period.

	Hgb (g/L)	CRP (mg/L)	L (/mm^3^)	AST (U/I)	ALT(U/I)
On admission	69	211	18,500	612	432
After 24 h (6 RCPs; embolization indicated)	75	223	19,300	623	441
After 48 h (2 more RCPs)	88	311	21,400	611	332
After 72 h (2 more RCPs; surgery indicated)	71	308	17,800	648	339
1st POD	92	188	16,500	580	311
3rd POD	102	92	15,300	498	294
7th POD	108	40	13,500	359	288
Discharge (POD 11)	118	22	1,1200	242	211

*RCP, Red cell packs; POD, postoperative day; Hgb, hemoglobin; CRP, C-reactive protein; L-leukocytes.*

## Discussion

Clinical presentation of HCC rupture may range from subtle, mild abdominal pain to acute abdomen and profound hemorrhagic shock. Computed tomography with intravenous contrast improved the accuracy of the HCC rupture diagnosis, however, in around 20% of the cases, the diagnosis is still made only during an emergency laparotomy.

The pathophysiological mechanism of spontaneous HCC rupture is still unclear and four possible factors may have a role as suggested by Sahu et al (15). Vascular injury hypothesis assumes that arteries supplying the tumor are becoming stiff and brittle which then rupture easily when tumor reaches subcapsular area. Venous congestion Hypothesis is based on pressure increase within the tumor and consequent intratumoral hemorrhages and necrosis. Similarly, the small room hypothesis suggests that the rupture happens when the tumor grows beyond its capacity, the inner pressure splits open the surrounding parenchyma and tears the capsule. It explains why most ruptured HCCs were sized more than 5 cm. Lastly, certain conditions (cirrhosis, portal vein thrombosis, and hypertension) and previous treatments (TACE, chemotherapy) were found to be associated with high HCC rupture risk.

Studies suggest that portal vein thrombus may be found in 12%–39% of patients with HCC and 18%–57% of patients with ruptured HCC (16–19). Although Yamada et al. (20) reported that TAE was contraindicated for patients with portal vein tumor thrombosis, due to the risk of ischemic liver damage. However, others have reported that TAE was a safe treatment method of HCC with peripheral portal vein tumor thrombosis given that the patient has a good liver function (21–23).

HCC rupture presents a great diagnostic and therapeutic challenge and treatment include a spectrum of modalities: from observation and conservative therapy using blood transfusions and coagulation corrections to radiologic or surgical intervention. The choice of treatment depends mainly on the hemodynamic stability of the patient and presence of active arterial bleeding. However, other factors should be considered such as tumor stage, the presence of coagulopathy, liver condition and patient's performance status (24). Pyogenic liver abscess may be caused by multiple processes and is usually polymicrobial. Most common causes are biliary tract infectious diseases in 60%, or hematogenous spread in 23% of the cases. Cryptogenic liver abscess accounts for 18% of the cases and other causes such as direct abscess spread, trauma or secondary tumor infections are rare (25). An untreated hepatic abscess may lead to complications that include sepsis, empyema, or peritonitis due to rupture into peritoneal spaces or retroperitoneal extension. Treatment should include drainage, either percutaneous or surgical. In general, abscess with size <5 cm are optimal for percutaneous drainage under CT or ultrasound guidance. Larger abscesses or those located near major hepatic vessels should be drained surgically. In both cases, antibiotic therapy is indicated from the admission and corrected after the results of the microbiologic analysis (26). There is only one case in the literature describing pyogenic liver abscess rupture with subcapsular hematoma. Authors suggest that such a scenario may mimic HCC rupture, but the tumor was not found in their case (27). In our case, however, HCC presence was obvious due to specific CT features and previous medical history.

To date, no clear guidelines have been proposed regarding the management of ruptured HCC. Therefore, it poses a significant therapeutic dilemma concerning the appropriate type and timing of intervention. Conservative treatment is possible in 22% of ruptured HCCs, but it requires close monitoring in the intensive care unit and carries a risk of recurrent bleeding. One-third of patients are treated with emergency embolization and another third with surgical exploration and hemostasis. The definite treatment of HCC after addressing the acute life-threatening condition should be discussed after reevaluation of the patient's condition and disease stage (28).

It should be emphasized that after resection, HCC tends to recur locally, therefore, if an HCC recurrence is discovered early, more therapeutic options may be available for the treatment of the recurrence. As such, close surveillance with cross-sectional imaging and *α*-fetoprotein measurement every 3–4 months for 2 years after curative intent is indicated and then every 6–12 months after this period (29). Ultrasound may be an unreliable screening for HCC recurrence due to low sensitivity. In our case, the patient has chosen ultrasound as a surveillance method despite recommendations for a CT scan given by the oncologist.

The exact etiology of portal vein thrombosis and liver abscess in our case remains unclear, but paraneoplastic syndrome, infected tumor or hematogenous spreading of distant septic focus are possible explanations. As mentioned above, portal vein thrombosis is commonly associated with HCC, unlike liver abscess which is not usual finding in HCC rupture. Subcapsular hematoma was probably a result of HCC rupture with venous bleeding and liver abscess could have been result of infected intrahepatic hematoma. Less probable, an abscess as initial incident, could have triggered HCC rupture. In addition, portal vein thrombosis may have caused ischemic liver changes, raised portal pressure, and consequent HCC rupture.

## Conclusion

The present study reports the case of a patient with a spontaneously ruptured recurrent HCC who had some of the aforementioned underlying factors (portal vein thrombosis and previous chemotherapy) however, the concomitant presence of liver abscesses, hematoma, simple cysts and portal vein thrombosis makes this case unique in the medical literature. Clinicians should be aware that multiple focal liver lesions of various origin may be concomitantly present within liver parenchyma and proper differentiation of each lesion is required for optimal therapy.

## Data Availability

The original contributions presented in the study are included in the article/Suplementary Material, further inquiries can be directed to the corresponding author/s.
